# What Sways People’s Judgment of Sleep Quality? A Quantitative Choice-Making Study With Good and Poor Sleepers

**DOI:** 10.1093/sleep/zsx091

**Published:** 2017-05-19

**Authors:** Fatanah Ramlee, Adam N. Sanborn, Nicole K. Y. Tang

**Affiliations:** 1 Department of Psychology, University of Warwick, Coventry, UK;; 2 Department of Psychology and Counselling, Sultan Idris Education University, Perak, Malaysia

**Keywords:** Sleep quality, subjective meaning, definition, mood, daytime functioning, insomnia, sleep, CBT.

## Abstract

**Study objectives:**

We conceptualized sleep quality judgment as a decision-making process and examined the relative importance of 17 parameters of sleep quality using a choice-based conjoint analysis.

**Methods:**

One hundred participants (50 good sleepers; 50 poor sleepers) were asked to choose between 2 written scenarios to answer 1 of 2 questions: “Which describes a better (or worse) night of sleep?”. Each scenario described a self-reported experience of sleep, stringing together 17 possible determinants of sleep quality that occur at different times of the day (day before, pre-sleep, during sleep, upon waking, day after). Each participant answered 48 questions. Logistic regression models were fit to their choice data.

**Results:**

Eleven of the 17 sleep quality parameters had a significant impact on the participants’ choices. The top 3 determinants of sleep quality were: *Total sleep time*, *feeling refreshed (upon waking*), and *mood (day after*). Sleep quality judgments were most influenced by factors that occur *during sleep*, followed by feelings and activities *upon waking* and the *day after*. There was a significant interaction between *wake after sleep onset* and *feeling refreshed (upon waking*) and between *feeling refreshed (upon waking*) and question type (better or worse night of sleep). Type of sleeper (good vs poor sleepers) did not significantly influence the judgments.

**Conclusions:**

Sleep quality judgments appear to be determined by not only what happened during sleep, but also what happened after the sleep period. Interventions that improve mood and functioning during the day may inadvertently also improve people’s self-reported evaluation of sleep quality.

**Statement of Significance**

Sleep quality is an elusive concept: there is currently no consensus definition of sleep quality. Using a choice-based conjoint analysis, this study was the first to quantitatively investigate the relative importance of 17 possible parameters (e.g., total sleep time, mood the day after) in sleep quality judgments. The study additionally shed new light on the interactions between parameters. The effects of parameter timing (day before, pre-sleep, during sleep, upon waking, day after), of existing sleep status (good vs poor sleepers), and of question type (which describes a better (or worse) night of sleep?) were also examined. The choice-based conjoint analysis represents a novel, and yet potentially more ecologically valid, methodology for uncovering the important parameters that define self-reported sleep quality.

## INTRODUCTION

Sleep quality is an important indicator of health and wellbeing in both healthy and clinical populations.^1–4^ In the context of sleep treatment, it is also an important patient-reported outcome used to reflect treatment progress or to determine treatment success.^5–8^

However, sleep quality is an elusive construct that is difficult to measure. Thus far, there is no consensus on the definition of sleep quality and what it consists of.^9^ Whilst it is understood that numerous factors related or unrelated to sleep can affect people’s judgment of sleep quality,^10,11^ little is known about their relative importance and how they interact with each other to sway sleep quality judgment.

Current measurements of sleep quality range from single-item rating scales to multi-item questionnaires. These various instruments and measurement approaches reflect their respective ideas of what sleep quality is all about. In terms of utility, each has their strengths but also their limitations. Single-item sleep quality rating scales (e.g., “how would you rate the quality of your sleep?”) are often used in daily sleep diaries^12^ and large-scale epidemiological studies.^13^ They provide a quick and yet undefined measurement of sleep quality since definitions of sleep quality do differ between individuals. In fact, one question often raised by patients/participants regarding this item on the sleep diary is ironically “what do you mean by sleep quality?”. In receipt of such question, the clinician’s/researcher’s spontaneous interpretation of sleep quality could have a strong influence over the patient’s/participant’s assessment of sleep quality. However, the simplicity of these single-item rating scales is attractive, as they are easy to use in clinical settings as well as research studies that require repeated measurements of daily sleep quality over a period of time. Multi-item questionnaires such as the Pittsburgh Sleep Quality Index (PSQI),^14^ and Medical Outcomes Study (MOS) sleep scale^15^ are well-validated measures of sleep quality and commonly used in research and clinical settings. In these instruments, sleep quality is represented by a composite score encompassing various aspects of (1) sleep experience during the night (e.g., sleep latency, sleep duration), (2) reports of sleep disturbances (e.g., waking up in the middle of the night, having to get up and use the bathroom, coughing or snoring loudly, having pain), (3) self-reported evaluation of sleep quality (e.g., good or bad, quiet or restless, feeling rested upon waking or not), (4) the bedroom environment (e.g., sleep disturbance from a bed partner or roommate, too hot or too cold), and (5) sleep-related behavior during the day (e.g., trouble staying awake, having to take sleep medication, having to take naps). These multi-item measures are comprehensive, but sleep quality is predefined for the respondents and may have been conflated with symptoms of sleep disorders (e.g., sleep apnea). Implicitly, these measures also assume that the respondents would put equal emphasis on each predefined factor while forming their overall judgment of sleep quality, which is at odds with the suggestion that different individuals tend to have different interpretations of what sleep quality is.^16^

A number of previous studies have attempted to identify physiological correlates of sleep quality. These physiological indices include both micro and macro measures of sleep architecture, such as cyclic alternating pattern rate,^9,17^ slow-wave sleep,^18^ percentage of REM,^19^ delta NREM EEG activity,^20^ sleep continuity/efficiency,^21^ number of awakenings at night,^22^ and total sleep time.^23^ Whilst these objective indices provide information about the possible physiological underpinning of the sleep experience, they do not correlate well with self-reported ratings of sleep quality^24,25^ and there are conflicting findings as to which objective measure is central to the self-reported judgment of sleep quality. For example, Landis et al.^23^ found that objective total sleep time was strongly correlated with sleep quality (*r* = .635, *p* < .01), whereas Westerlund et al.^18^ demonstrated that the amount of time spent in stage 2 sleep, not objective total sleep time, was the only significant predictor of sleep quality (β = −.07, *p* < .01).

More recently, there is a renewed interest in investigating the definition of sleep quality from the sleeper’s perspective using qualitative approaches. This qualitative approach acknowledges sleep as a private, subjective experience and has proved to be a particularly fruitful method for collecting in-depth data from individuals based on their interpretations of what happened during, as well as before and after, sleep. Using focus group discussion, Kleinman et al.^26^ explored the language 28 patients with insomnia used to describe their sleep experience. Discussions were also generated based on descriptions written by the patients, who were asked to write down words that described to them a “good night’s sleep”. Some of the phrases used to describe a good night’s sleep were “restful”, “peaceful”, “sound sleep”, and waking up feeling “refreshed”, “energetic”, and “motivated”, whereas a poor night’s sleep was typically characterized by physical and cognitive “restlessness” as well as waking up feeling “tired” and “exhausted”. Meanwhile, Harvey and colleagues^27^ asked participants with insomnia and normal sleepers to talk freely for 3 minutes about the characteristics of a night when they experienced good sleep quality and then for another 3 minutes about poor sleep quality. These authors combined this speak freely procedure with a semistructured sleep quality interview and a week’s worth of sleep diary to examine the subjective meaning of sleep quality. From their mixed-methods analysis, they found that—to the participants—sleep quality was most commonly defined by “tiredness on waking and throughout the day”, “feeling rested and restored on waking”, and “number of awakenings experienced in the night”. Interestingly, in their analysis of the meaning of sleep quality they found that both people with insomnia and normal sleepers had similar definitions of sleep quality, although people with insomnia tended to use more criteria to judge a good night’s sleep than the normal sleepers. Taken together, findings from Kleinman et al.^26^ and Harvey et al.^27^ reveal that people use multiple criteria to judge their sleep quality, and that the factors affecting the judgment of sleep quality can occur during the night as well as beyond the typical nighttime sleep period. There appears to be some systematic differences between good and poor sleepers in the way in which they judge the quality of a good night’s sleep and poor night’s sleep, but further investigation is required to confirm these main effects.

The present study aimed to extend our understanding of the factors influencing our sleep quality judgment, by examining the relative weights they carry in the sleep quality judging process. We were also interested in examining the possible interaction between the parameters of sleep quality extracted from different time periods, between different types of sleeper, and between different types of judgment. To do so, we conceptualized the self-report sleep quality as a decision-making process. By that, we mean, when people make judgment of their sleep quality, they will inevitably have to process and integrate their memories of their sleep experience during the night, their feelings on waking, and their assumed impact of sleep quality on their functioning the next day. People will have to weigh up the relative importance of the various factors/criteria that make up their good or poor night’s sleep. For example, in our research with people with chronic pain and comorbid insomnia, we found that patients considered pain and discomfort in the morning and how much they can physically do during the day as the most important indicators of sleep quality of the night before.^28^

Once we had conceptualized the sleep quality judgment as a decision-making process, we saw that the challenge of finding the factors that led to a good and poor night’s sleep was similar to the challenge of product design and marketing. Product managers need to determine how consumers weigh various factors, such as screen size and resolution, when evaluating the quality of a television. Whilst we are interested in sleep quality instead of television quality, and the factors that can influence sleep instead of screen size and resolution, these problems are essentially the same. Thus, we can use methodology commonly deployed to address this question: choice-based conjoint analysis. In choice-based conjoint analysis, individuals are presented with a series of choices between options with different features, and regression models are used to infer how the features are weighted.^29,30^ Choice-based conjoint analysis represents a novel, and yet potentially more ecologically valid, methodology for uncovering the important parameters that determine people’s sleep quality judgment.

## METHODS

### Design

In our choice-based conjoint analysis, instead of evaluating each sleep parameter individually, we simulated the real-life decision-making process by presenting our participants with 2 concrete descriptions of sleep/wake scenarios comprising a combination of sleep quality parameters highlighted in the literature. After repeating this choice exercise over a sufficient number of trials, we used regression to quantitatively estimate the relative importance of all included parameters of sleep quality and examined if these parameters interact with each other.

A quantitative choice-making study was thus conducted with 100 young adults. In the first part of the study, the participants were asked to complete a set of questionnaires, which contained items asking about the participant’s demographics, typical sleep pattern, and insomnia severity in the past 3 months. These data were used to characterize the participants. The second part of the study was an experimental session, during which the participants were asked to read and choose between 2 scenarios to answer the question “Which describes a better night of sleep?” in half of the trials, or “Which describes a worse night of sleep?” in the remaining half of the trials. Each scenario described a self-reported experience of sleep, in the first person narrative, stringing together 17 possible determinants of sleep quality that we had identified from our literature review. These determinants are referred to below as “sleep quality parameters” or “parameters of sleep quality judgment”. Each participant answered 48 questions (i.e., 48 trials) and the data from these trials were used to evaluate the relative importance of each sleep quality parameter.

The protocol of the study received full ethical approval from Humanities and Social Sciences Research Ethics Committee (Reference number: 44/13–14), University of Warwick. All participants were paid an honorarium for their participation.

### Participants

Participants of this study were aged between 18 and 30 years and were recruited from a university-wide subject panel. The study was conducted with young adults to minimize the effect of comorbid psychiatric and medical symptoms and the use of medications on decision-making.^10^ Participants were included in the study if they were (1) aged 18 to 65 at the time of the study and (2) English-speaking. Participants were allocated to the “good sleeper” group if they scored 7 or below on the Insomnia Severity Index (ISI)^31^ indicating no clinically significant insomnia. Participants were included in the “poor sleeper” group if they scored 8 or above on the ISI and had been experiencing 1 or more of the following symptoms for at least 3 nights a week for at least 3 months, despite having an adequate opportunity to sleep: (1) difficulty initiating sleep (taking longer than 30 minutes to fall asleep), (2) difficulty staying asleep (frequent midnight awakenings), (3) early morning awakening with an inability to return to sleep, (4) daytime functioning impairment (e.g., poor concentration, excessive sleepiness). These insomnia symptoms were assessed using a self-report checklist and these group allocation criteria were set in line with the DSM-5 diagnostic criteria for insomnia disorder, for assessing the presence of insomnia symptoms.^32^

**Figure 1:**
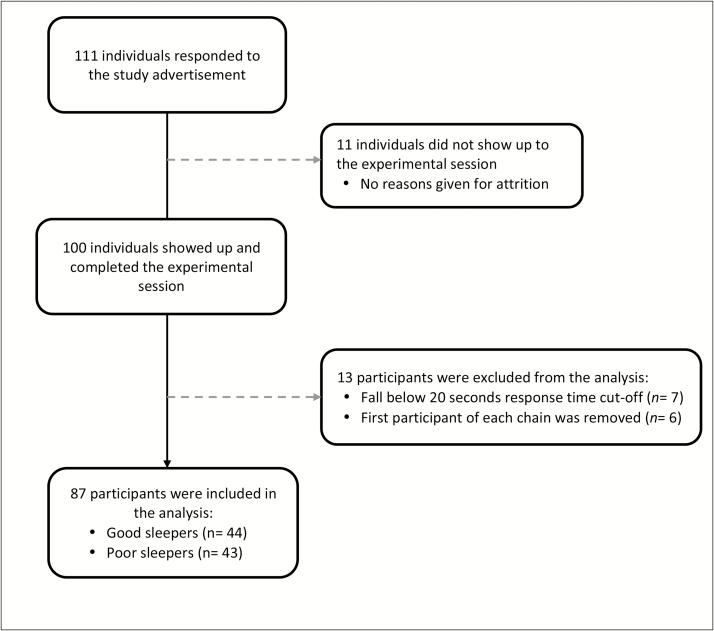
Flow diagram of participant recruitment.

Figure 1 shows the recruitment flow diagram. Of the 111 individuals who responded to the recruitment advert, 50 participants met the criteria of good sleeper, 50 participants met the criteria of poor sleeper, and the remaining individuals did not show up to the experimental session (*n* = 11). Seven participants were excluded from the analysis on the basis of an average trial completion time of less than 20 seconds, which was an extremely fast completion time that suggested noncompliance to the task instruction. This cut-off completion time was determined based on the pilot study (see Materials section in Methods). A further 6 participants were excluded due to the methodological necessity to remove the first participants of each data chain (see the Analysis section for an explanation).

### Procedure

Potential participants who responded to the recruitment advert completed a screening/demographic questionnaire and attended an experimental session. Written informed consent was obtained from the participants before commencing the experimental session, which took place in small groups of 3 to 4 participants in a lab with multiple computers partitioned into stations. The lab was sound attenuated with central air conditioning and lighting control. Each participant was assigned to a computer at some distance from the others to minimize distraction and response contamination.

The participants were asked to read and imagine themselves being the person experiencing 48 pairs of scenarios. They read a pair of scenarios in each trial and were asked to choose one scenario from each pair that represents a night of better (or worse) sleep quality, depending on the question that they were presented. To avoid misunderstanding what was being expected from the tasks, in addition to verbal explanations the participants were given detailed written instructions on the computer screen (see Supplementary Appendix 1).

**Figure 2:**
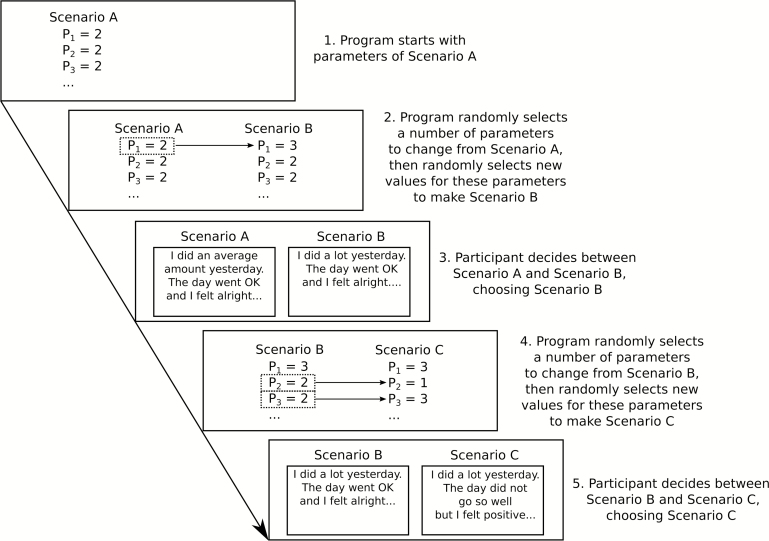
An example of how the MCMCP algorithm mutates the scenarios in response to a participant’s choices. The notation P_*x*_ = Y refers to parameter *x* in Table 1, which is set to level Y. For example, P_1_ = 2 means that the first parameter, “amount of activity”, is set to the second level, “an average amount”. MCMCP = Markov Chain Monte Carlo with People.

The number of trial for each participant was set to 48 due to concerns of task fatigue and the practical time limits of reading speed, which does not allow for the comparison of the huge number of possible stories (3 options ^16parameters^ × 5 options ^1 parameter^ = 215 233 605 stories). Instead, we aimed to present a subset of stories that were similar enough to be easily comparable, and also to focus on stories that would lead to the most extreme ratings of sleep quality. Previous work in conjoint analysis has used genetic algorithms for the task, as genetic algorithms work by presenting a large set of candidate scenarios, from which the participant selects those that will “survive”. The surviving scenarios are mutated to produce new options, and the process repeats until a good set of candidate options has “evolved”.^33^ However, because participants will likely become confused when choosing amongst a large number of scenarios, we used a simpler algorithm with the same properties, Markov Chain Monte Carlo with People (MCMCP;^34^ see details in Supplementary Appendix 2), which accomplishes the same goal by presenting choices between pair of scenarios. Similar to a genetic algorithm, after the participant has made a choice, the chosen scenario is “mutated” to produce a new scenario, and the participant then decides whether their previous choice or the new scenario is better. Scenarios were mutated by changing a random number of parameters, which was drawn from a truncated geometric distribution with a mean of number of parameter changes of 4.6. Each parameter was equally likely to be changed, and all new options for a parameter were equally likely. Figure 2 graphically depicts the scenario mutation process and the corresponding actions required from the participant at each stage.

The sequential nature of MCMCP means that the scenarios are chained together, with the previous choice influencing what is presented on the next trial. Because we did not wish to make the sequential nature of the trials obvious, we created multiple independent chains which were interleaved together.

The 48 trials were presented in 4 chains of 12 trials; 2 chains asking participants “Which describes a better night of sleep?” and another 2 chains asking “Which describes a worse night of sleep?”. Also, the chains carried over from 1 participant to the next: this enhanced the power of the analysis at the cost of assuming no individual differences in how participants weighted the factors.^35^ Finally, to improve the speed of data collection we set up multiple groups of chain that could be run in the same testing session: 3 good sleeper and 3 poor sleeper groups (see Supplementary Appendix 3).

Each testing session was approximately 50-minute long. Participants were given a 5-minute indoor break after 24 trials to counteract any task-related fatigue. However, no stimulant use (coffee, tea, energy drinks, or cigarettes) was allowed during the break. All participants completed the task and were paid an honorarium at the end of the testing session.

### Materials

As shown in Table 1, each sleep scenario contained 17 adjustable parameters. These parameters were chosen following a review of relevant studies that examined the factors that influence people’s judgment of sleep quality.^16,18,21,26,27,36–38^ The selection of parameters was also informed by themes that emerged from a recent qualitative study conducted by our group, in which we explored the criteria people use to judge their sleep quality.^28^ In this study, we found that people by and large rely on their (1) memories of nighttime sleep disruptions, (2) feelings on waking and cognitive functioning during the day, (3) ability to engage in daytime physical and social activity, and (4) changes in physical symptoms as key criteria for evaluating their sleep quality. Accordingly, the chosen parameters were not restricted to the sleep period, but included factors that spanned from the day before the sleep period to the day after. The selection of parameters was led by the last author (NT; who has clinical and research experience working with individuals with and without insomnia), in consultation with the first author (FR) regarding the content and with advice from the second author (AS) regarding the feasibility and programmability of the scenarios and computing resources required. Disagreements were resolved by team discussion. Parameters retained after discussion were then tested in a pilot study with 64 young adults, which helped us to identify programming errors, readability of the resultant scenarios, speed of reading, compliance to the instructions, and efficiency in generating distinguishable scenarios for analysis (i.e., number of burn-in trials).

**Table 1:** Options for each parameter of sleep quality.

Parameters	Option 1	Option 2	Option 3	Option 4	Option 5
Day before					
Amount of activity	I did little	I did an average amount	I did a lot		
Day went well?	Did not go so well	Went OK	Went well		
Mood	I felt rubbish	I felt alright	I felt positive		
Pre-sleep					
Readiness to sleep	I did not feel sleepy at all	I felt moderately sleepy	I felt very sleepy		
Cognitive arousal	My mind was racing with thoughts	My mind was wandering with thoughts	My mind was blank		
Physiological arousal	I felt very uncomfortable	I felt not so uncomfortable	I felt very uncomfortable		
During sleep					
Sleep onset latency	It took me a long time	It took me a short while	It took me no time		
Wake after sleep onset	I woke up in the middle of the night and was unable to fall back to sleep	I woke up in the middle of the night and was eventually able to fall back to sleep	I woke a number of times but only briefly	I woke once or twice but only briefly	I slept through the night
Total sleep time	I think I slept for 9.5 hours	I think I slept for 7.5 hours	I think I slept for 5.5 hours		
Dream	I remember having many dreams	I remember I dreamt	I don’t remember any dreams		
Upon waking					
Feeling refreshed	I felt unrefreshed	I felt somewhat refreshed	I felt refreshed		
Motivated to get up	I felt unmotivated	I felt somewhat motivated	I felt motivated		
Day after					
Alertness	I felt drowsy	I felt tired	I felt alert		
Thinking	My head felt cloudy	My head was reasonably clear	My head was clear		
Mood	My mood was bad	My mood was average	My mood was good		
Sociability	I was antisocial	I was somewhat sociable	I was sociable		
Physical activity	I was sluggish	I was reasonably active	I was active		

The 17 final parameters were weaved together with predetermined pronouns and conjunctive words/phrases to generate a first person account of sleep experience. Each parameter had 3 options, with the exception of “*wake after sleep onset*” (*WASO*) for which 5 options were provided. The combination of parameters and options allows us to generate 215233605 possible scenarios.

**Figure 3:**
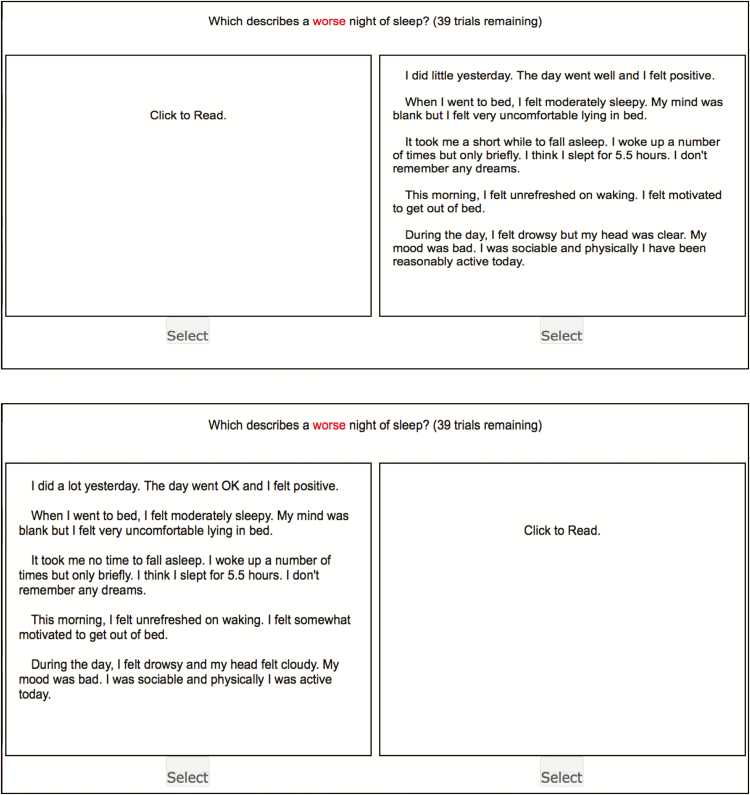
Example of scenarios that were presented to a participant. “The upper panel” was the first scenario that appeared on screen; “The lower panel” was the second scenario appeared on screen; “Scenario”: each set of sleep scenarios presented in the box comprised 17 adjustable sleep quality parameters; “Click to Read”: participant used this button when s/he was ready to read the scenario; “Select”: participant used this button to indicate his/her choice; “Types of question”: Which describes a worse night of sleep?; “Trials”: Each participant had 48 trials to complete, for examples, “39 trials remaining” in the above example means the participant had finished 9 trials and had 39 trials remaining.

Figure 3 presents a screen-shot of 2 example scenarios from which the participants had to select one of the scenarios to answer the question, “Which describes a worse night of sleep?” Only one scenario was visible at a time and the scenario could not be viewed again after it had been viewed twice. Viewing was restricted in this way in order to encourage the participants to make a sleep quality judgment based on their imagination of the scenario as a whole, instead of making their judgment based on comparisons of individual parameters between scenarios. We never asked participants to report these holistic impressions, but instead just asked them to use these internal impressions to make a choice between the 2 scenarios. We observed the choices, and used a logistic regression analysis to determine how each parameter was weighted when making these choices.

### Analysis

Data were analysed using the statistical software R (http://www.r-project.org/). Descriptive statistics were used to describe participants’ characteristics. Means and standard deviations were presented to describe continuous variables, whilst frequencies and percentages were reported for categorical variables. Independent sample *t*-test and chi-square statistics were used to describe the differences in characteristics between the good and poor sleeper groups.

Chains were first analysed to determine the best number of trials to discard as burn-in trials (i.e., choices that had not yet “evolved” into good or poor sleep quality scenarios). Analyses using the Brooks and Gelman^39^ convergence diagnostic indicated that it was best to remove very few trials, so only the first participant was removed from each chain. As a result, 6 participants were excluded from the analysis in addition to 7 participants who were excluded because they fell below the 20 seconds cut-off response time during the trials.

The effect of each parameter on choices made was examined using logistic regression. The logistic regression model was performed on all of the data including both questions (“Which describes a better night sleep?”; “Which describes a worse night sleep?”) and both good and poor sleepers. The data were drawn from 87 participants who completed 48 trials each, which produced 4176 choices.

The dependent variable was which scenario was judged to be a better night’s sleep for both types of question asked. The parameters found in the logistic regression are interpretable as log odds: they quantify how much more or less likely a participant would choose a scenario if a particular option is included.

The logistic regression model included main effects of each parameter as well as a variety of interactions. Specifically, the terms of the model are:

1. The 17 parameters listed in Table 1 (e.g., *mood*, *sleep onset latency* [*SOL*], *physical activity*)
2. Two-way parameter interactions. These were the interactions between the 4 parameters *during sleep* (*SOL*, *wake after sleep onset* [*WASO*], *total sleep time* [*TST*], and *dream*) crossed with the 7 *upon waking* and the *day after* parameters (feeling *refreshed, motivated to get up, alertness, thinking, mood, sociability,* and *physical activity*), yielding 28 terms out of the possible 272 pairwise interactions between parameters. These interactions were selected because better experiences the next day were expected to mitigate a poorer night’s sleep.^40,41^
3. Two-way interactions between parameters and types of sleeper (e.g., *SOL* and good sleepers), which added an additional 17 terms.
4. Two-way interactions between parameters and types of question (e.g., *alertness* and *Which describes a better night of sleep?*), which added an additional 17 terms.

Statistical tests were performed by comparing the full model to restricted models that did not include a parameter or any higher-level interactions with that parameter. For example, when assessing whether *WASO* was a significant determinant of sleep quality judgment, the full logistic regression model was compared to a restricted model without the *WASO* term or any interactions that included *WASO*. This approach to jointly test whether a parameter has an effect by comparing a full model to a model with both the interaction and the main effect removed has been proposed for use in genetics by researchers who are interested in whether a gene has either a main effect or an interaction with the environment, but are unsure which one it will be.^42^ Nested models were compared using likelihood ratio tests, where the difference in deviances of the models is compared against a chi-squared distribution with degrees of freedom equal to the difference in number of parameters of the 2 models. The type I error rate was set to 0.01 to control for multiple comparisons.

## RESULTS

### Participant Characteristics

Table 2 presents the participants’ characteristics by group. The mean age of the 87 participants included in the analysis, 60% female, was 22.5 years. There were significant differences between good and poor sleeper groups on the ISI and other sleep variables. The good sleeper group scored lower on the ISI, awoke less often, had shorter WASO, took less time to fall asleep and had greater TST than the poor sleeper group. There was no difference between the good and poor sleeper groups in terms of their age, BMI, sex, ethnicity, and first language.

**Table 2:** Participant’s sleep and demographic characteristics.

	Group total	Good sleeper	Poor sleeper	Comparison between good and poor sleeper
	*n* = 87	*n* = 44	*n* = 43	
Demographic variables				
Age (years)	22.5 (2.6)	22.6 (2.6)	22.3 (2.6)	*t*(85) = .68
BMI	21.7 (3.3)	21.7 (2.8)	21.8 (3.8)	*t*(76.96) = −.14
Sex				
Male	35	20	28	*χ* ^**2**^ (1, *N* = 87) = 3.3
Female	52	24	15	
Ethnic origins				
White	27	16	11	*χ* ^**2**^ (1, *N* = 87) = 93.3
White Irish	1	1	0	
Asian British: Chinese	30	14	16	
Asian British: Indian	10	7	3	
Asian British: Asian other	15	4	11	
Black or Black British	1	1	0	
British mixed	1	0	1	
Other	2	1	1	
First language				
English	38	20	18	*χ* ^**2**^ (1, *N* = 87) = 1.4
Other	49	24	25	
Sleep variables				
ISI	8 (5.3)	3.61 (2.1)	12.5 (3.5)	*t*(68.35) = −14.43***
Typical SOL (mins)	24.9 (24)	15 (14)	35.12 (27)	*t*(62.35) = −4.24***
Typical WAKE (mins)	1	1	2	*t*(56.64) = −3.52**
Typical WASO (mins)	6.5 (10.4)	3.2 (6.4)	9.9 (12.5)	*t*(62.23) = −3.2***
Typical TST (mins)	457 (77.1)	483 (78.7)	430 (66.5)	*t*(85) = 3.36***

Mean values are presented with standard deviations in parentheses, except for sex, ethnicity, and first language where the number of count (frequency) is presented. BMI = body mass index; ISI = Insomnia Severity Index; SOL = sleep onset latency; TST= Total sleep time; WAKE = number of wake after sleep onset; WASO = wake after sleep onset.

*p < .05; **p < .01; ***p < .001.

### Effects of Individual Parameters on Sleep Quality Judgment

**Figure 4:**
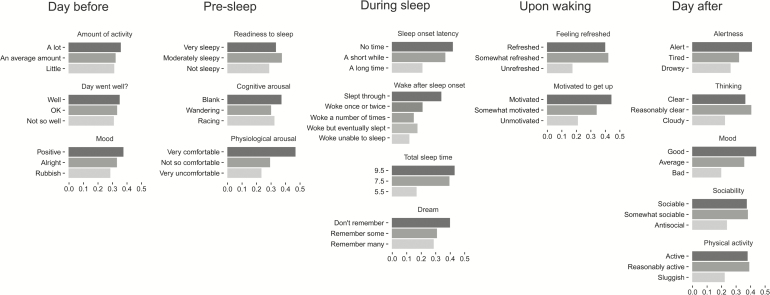
Descriptions of a good night’s sleep. Seventeen adjustable sleep quality parameters (bar plots) and their options (individual bars) are organized by 5 time periods. The relative bar lengths of 2 options represent the relative probability of choosing a scenario that contains those options, for example, because the bar for “No time” for *sleep onset latency* is twice as long as the bar for “A long time”, then a scenario that contains “No time” is twice as likely to be chosen as a scenario that contains “A long time”, all other parameters being equal.

The multiple parameters of sleep quality covered the experience of the *day before* sleep, during the *pre-sleep* period, *during sleep*, *upon waking*, and the *day after* (see Figure 4). The parameters that occurred during the *day before* sleep did not have a significant impact on the participants’ choices (*amount of activity*: *p* = .38; *day went well?*: *p* = .93; *mood*: *p* = .19). Of the *pre-sleep* parameters, only *physiological arousal* (*p* < .001) had a significant impact on the participants’ choices (*readiness to sleep*: *p* = .06; *cognitive arousal*: *p* = .09). Of the *sleep* parameters, *SOL* (*p* < .001), *WASO* (*p* < .001) and *TST* (*p* < .001) had a significant impact, whereas memory of *dream* (*p* = .08) did not have a significant effect on the participants’ choices. Both of the *upon waking* parameters had a significant impact (*feeling refreshed*: *p* < .001; *motivated to get up*: *p* < .001). All of the *day after* parameters had a significant impact (*alertness*: *p* = .01; *thinking*: *p* < .001; *mood*: *p* < .001; *sociability*: *p* < .001; *physical activity*: *p* < .001).

An analysis was run to compare the importance of different individual parameters and different time periods in explaining the participants’ choices. This analysis was performed by fitting the choice data with single factor logistic regression models (e.g., a model using only the parameter *amount of activity*) and comparing how well each model fit the data. We performed this comparison using Bayesian Information Criterion (BIC), which is a method for trading off goodness of fit against model complexity.^43^ Better (i.e., lower) BIC values are given to models that explain the data well without too many parameters. Using this measure, as shown in Table 3, the most important individual parameter of sleep quality, was *TST*, followed by *feeling refreshed* (*upon waking*), then *mood* (*day after*) and then *motivated to get up* (*day after*). We also performed the same analysis on time period by fitting the choice data with logistic regression models that included all parameters within a single-time period (e.g. the model for *upon waking* had both the *motivated to get up* and *feeling refreshed* parameters). As shown in Table 3, the most important time period was *during sleep*, followed by *upon waking*, then *day after*, then *pre-sleep*, and finally the parameters that occurred *day before* sleep were least important.

**Table 3:** Individual parameters and time periods log likelihood and BIC values.

	Log likelihood	BIC values
Parameters		
Total sleep time	−2822	5668
Feeling refreshed	−2824	5674
Mood (day after)	−2845	5714
Motivated to get up	−2851	5727
Wake after sleep onset	−2846	5733
Sleep onset latency	−2858	5741
Physiological arousal	−2863	5751
Physical activity (day after)	−2865	5755
Thinking (day after)	−2868	5762
Alertness (day after)	−2875	5775
Sociability (day after)	−2877	5780
Mood (day before)	−2880	5784
Dream	−2885	5795
Readiness to sleep	−2886	5797
Cognitive arousal	−2888	5802
Amount of activity (day before)	−2889	5803
Day went well? (day before)	−2891	5808
Time period		
During sleep	−2740	5573
Upon waking	−2788	5617
Day after	−2766	5624
Pre-sleep period	−2854	5766
Day before	−2876	5810

The log likelihood (larger is better) and BIC values (smaller is better) combine goodness of fit with a penalty for complexity. Parameters and time periods are ordered from most to least important (i.e., by BIC values). BIC = Bayesian information criterion.

Based on the log odds estimated for each significant parameter option, the “best-preferred scenario” for a better night’s sleep was as follows, with words in bold/italic indicating the adjustable option of the 11 significant parameters:

“I felt ***very comfortable*** lying in bed. It took me ***no time*** to fall asleep. I ***slept through the night***. I think I slept for ***9.5******hours***. This morning, I felt ***somewhat refreshed*** on waking. I felt ***motivated*** to get out of bed. During the day, I felt ***alert*** and my head was ***reasonably clear***. My mood was ***good***. I was ***somewhat sociable*** and physically I was ***reasonably active*** today”.

### Interaction Between Parameters of Sleep Quality

We examined the interactions between the 4 parameters *during sleep* (*SOL*, *WASO*, *TST*, and *dream*) crossed with 7 *upon waking* and the *day after* parameters (*feeling refreshed*, *motivated to get up*, *alertness*, *thinking*, *mood*, *sociability*, and *physical activity*). Of the pairwise interactions between these parameters, only *WASO* and *feeling refreshed* had a significant interaction (*p* < .001). This interaction judged a night with both *WASO* and *feeling unrefreshed* to be a particularly poor night’s sleep. However, if participants either felt at least *somewhat refreshed* or if they *slept through* the night, then they judged it to be a reasonably good night’s sleep.

### Interactions Between Parameters of Sleep Quality Judgement and Types of Sleeper

There was no significant interaction between parameters and types of sleeper, suggesting that whether the participant was a good or poor sleeper did not have a significant effect on their choices.

### Interactions Between Parameters of Sleep Quality Judgment and Types of Question

The interaction between parameters and types of question allowed us to statistically test whether participants used the same parameters to define a good and a bad night’s sleep. Only one significant interaction was found between *feeling refreshed* and types of question (*p* = .003), suggesting that *feeling refreshed* was more important to the participants when judging a good night’s sleep than when judging a poor night’s sleep.

## DISCUSSION

Instead of asking people to give an abstract rating of their sleep quality, we asked people to make choices between 2 concrete scenarios and indicate with their choice which scenario represents a better (or worse) night’s sleep. By conceptualizing the sleep quality judgement as a decision-making process, we managed to quantitatively identify and estimate the relative importance of different sleep and nonsleep parameters in influencing their judgement of sleep quality. In this study, 11 out of 17 identified sleep quality parameters were found to have a significant effect on the participants’ sleep quality judgment. In particular, the participants relied most heavily on *TST*, *feeling refreshed* (upon waking) and *mood* (day after) to make their judgment of sleep quality. The data also suggested that the participants’ judgment of sleep quality was most influenced by their memories of what happened *during sleep* and their experience *upon waking*, followed by their feelings and functioning during the *day after*, then *pre-sleep* experience of the night before, and lastly their experience the *day before*. Synergetic effects were found (1) between *WASO* and *feeling refreshed* (upon waking); (2) between *feeling refreshed* (upon waking) and types of question. However, whether the participant was a good or poor sleeper did not appear to make a difference in the way in which the sleep quality judgment was made. Below we ponder several themes/questions emerged from the findings.

### Sleep Quality Judgment Is Influenced by Multiple Parameters Spanning Across Different Times of the Day

This may in part explain why the field has thus far unable to pinpoint what the defining feature of sleep quality is.^9^ Sleep is a behavioral state of reduced activity and people typically remember little of what happened during the hours of sleep.^44,45^ In contrast, the feelings they have upon waking and their evaluations of their own mood and daytime performance are relatively more accessible information. It is understandable why participants in the current study drew on both their memory of nighttime sleep and experience during the day to retrospectively judge their sleep quality. This combined use of day and night information for judging sleep quality resonates with previous work suggesting a significant role of daytime impairments in the genesis of insomnia complaint.^37,40,41,46–48^ The retrospective and inferential nature of the decision-making process also raises 2 interesting possibilities for future investigation. First, people’s judgment of sleep quality may vary depending on the time of the day the question is presented and the amount of relevant information accessible for retrieval when the judgment is called for. Second, the judgment of sleep quality can potentially be altered by systematically restructuring a person’s daytime experience or by reversing biases in their evaluation of their mood and daytime functioning.

In terms of the content of the information used, *TST*, *feeling refreshed* (*upon waking*) and *mood* (*day after*) were the top 3 parameters influencing the judgment of sleep quality. Interestingly, combinations of these top parameters bear striking similarity with some of the statements featured in the Dysfunctional Beliefs and Attitude about Sleep (DBAS),^49^ for example, “I need 8 hours of sleep to feel refreshed and function well during the day” and “By spending more time in bed, I usually get more sleep and feel better the next day”. It is possible that endorsement of rigid, unhelpful sleep beliefs can have a direct or indirect effect on people’s judgment of sleep quality.^40,41^ This effect may not be restricted to people with insomnia disorders but also apply to those experiencing other sleep disorders such as sleep apnea, restless legs syndrome, hypersomnia, or narcolepsy.^50^ We note though that in the “best preferred scenario” generated from the data of our participants, they indicated that they preferred 9.5 hours to 7.5 hours (which is closer to the typically expectation of 8 hours). This deviation may reflect the developmental sleep need of our participants whose mean age was 22.5 years at the time of the study.^51,52^

### Pre-Sleep Cognitive Arousal is not a Significant Parameter of Sleep Quality?

Of all pre-sleep parameters tested, only physiological arousal had a significant impact on the participants’ judgment of sleep quality. This is in contrast to the established understanding that poor sleepers refer to cognitive arousal rather than physiological arousal as the premise of their insomnia^53,54^ and that hyperarousal during the pre-sleep period—manifested either cognitively as worry/rumination or physiologically as high-frequency beta EEG—is a strong predictor of subsequent low sleep quality (see Riemann et al.^55^ for a review). The null finding of pre-sleep cognitive arousal may be explained by how it was operationalized in our current study. In the sleep scenarios, the options given to the participants were: my mind was “racing with thoughts”, “wandering with thoughts”, or “blank”. In retrospect, these choices only described the frequency of cognitive activity but not the tone of the cognitive activity. Potentially, a heightened amount of cognitive activity *per se* is not sufficient to alter people’s sleep quality judgment. It may be essential that the heightened amount of cognitive activity is negative or even threat-provoking in order to sway people’s perception of sleep quality.^41,56^

### WASO and Feeling Refreshed Are not Functionally Synonymous, but Interacting Parameters of Sleep Quality?

The interaction suggests that if the participants did not sleep through the night and did not feel refreshed in the morning, they would be disproportionately more likely to come to the conclusion that they had had a poor night’s sleep. However, if the participants somehow feel refreshed on waking, whether or not they have slept through the night would not be as important as it would normally be in their judgment of sleep quality. This finding raises the possibility that sleeping through the night may not be a prerequisite to feeling refreshed the next morning. The nonlinearity is possible because, like sleep quality, feeling refreshed is a nonspecific subjective judgment which may or may not be influenced by the sleep experience, post-sleep inertia and sensory input, and/or the person’s ability to look forward to activities/excitement lined up for the day. Exploring ways to help people feel “refreshed” in the morning could potentially provide a new route to improve sleep quality among people with mild-moderate sleep maintenance problems, and we would like to propose 2 plausible avenues: (1) introducing attentional training that helps people with insomnia to reverse or diffuse attentional biases toward negative, threat-provoking sleep cues^57,58^ and to apply heavier weights on positive memories and experience to inform their sleep quality judgment; (2) instead of focusing exclusively on nighttime experience, insomnia treatment may diversify to help patients regulate their physical and social activity during the day. Based on the findings of the current study, improved mood and perceived daytime functioning can influence a person’s overall sleep quality judgment.

### No Systematic Difference in the Way Good and Poor Sleepers Judge Their Sleep Quality

Although counterintuitive, this finding is consistent with the key observation from Harvey et al.,^27^ in which normal sleepers and people with insomnia used broadly similar characteristics to describe a good/poor night’s sleep when asked to define sleep quality or to explicitly state what is important for their judgment of sleep quality. Together, the findings from both Harvey et al.^27^ and our study appear to suggest that there are certain universal requirements for good-quality sleep shared between good and poor sleepers, and people with insomnia are not exaggerating their sleep quality requirements simply because of their distress or personal experience of sleeplessness. An intriguing question left unanswered is what sets these requirements? Are the requirements biological or socially determined through acculturation? Future anthropological studies comparing sleep quality parameters used by distinctive cultural groups with different sleep patterns and contexts may help address the question.^59^

### Strengths and Limitations

Several potential limitations of the current study should be discussed. First, the participants were young, generally healthy adults drawn from a university community. Such demographic background is restricted in diversity. Although the participants who had an ISI^31^ score of 8 or above and presented with insomnia symptoms that mapped onto the DSM-5 diagnostic criteria^32^ were allocated to the poor sleeper group, they may not represent patients diagnosed with insomnia who are actively seeking medical or nonpharmacological treatment. Generalizability of the findings to the wider clinical population with more severe insomnia symptoms is yet to be determined. In relation to this, given the encouraging results generated by this study, future study should also consider exploring the judgment of sleep quality in more heterogeneous sample with varied characteristics (e.g., older population, people from different socioeconomic spectra of the society, patients living with chronic medical/psychiatric conditions, etc.). Second, to maximize efficiency and statistical power of the study, administration of the choice-making task took place at different times of the day. Whilst we have tight control over the testing environment and the participants’ use of stimulants, exposure to light, amount of activity, and task-related fatigue during the testing period, we do not know to what extent the result could have been subject to the influence of circadian rhythm. To address this question, future follow-up studies may want to add measures of the participants’ alertness levels at the start of the task and time the testing session according to the participants’ circadian preference (e.g., morningness-eveningness).^60^ Third, to simulate the real-life decision-making process and to standardize the number of parameters used for making a sleep quality judgment, we asked the participants to read and imagine themselves being the person experiencing the sleep/wake scenarios, and then choose the one that best represents a better (or worse) night’s sleep. Whilst detailed instructions were given to the participants, the extent to which they successfully identified themselves with the scenarios was not certain, although we did eliminate from the analysis data of those participants who responded too quickly to have engaged with the task. Also, whilst we gained ecological validity by simulating the decision-making process through combining different parameters of sleep quality, the choice-making task was nonetheless presented on a computer screen in an artificial testing environment. Future studies should consider situating the choice-making task within real-life scenarios as hypothetical situations can cause participants to overestimate or underestimate the effect parameters have compared to actual experience,^61^ although sleep is arguably a near universal experience. It is fair to assume all participants have some degree of lived experience to support their imagination of good- and bad-quality sleep scenarios, which is not of the same degree of difficulty as though they were asked to imagine what quality of life they would have had they experienced paraplegia^62^ or had lost a limb to cancer.^63^ That said, the current study was the first to examine the parameters of sleep quality using a quantitative choice-making approach. Using this method, we managed to string together different parameters from different time periods and examined the effect of time, sleepers, and interaction between parameters in explaining participant’s sleep quality judgment. The sleep quality parameters were anchored to different concrete options (e.g., WASO: “I slept through the night.”, “I woke up once or twice but only briefly.”, “I woke up a number of times but only briefly.”, “I woke up in the middle of the night and was eventually able to fall back to sleep.”, “I woke up in the middle of the night and was unable to fall back to sleep”). This provided specific directions and a definition for each parameter in order to reduce differences in how participants interpreted parameters based on their previous individual sleep experiences. This is a methodological improvement over qualitative studies, interviews, questionnaires, and sleep diaries in extracting information relevant to sleep quality judgment. Readers with a statistical background may have noticed that there is a degree of correspondence between Item Response Theory (IRT) and choice-based conjoint analysis used in the current study. However, there is one important difference: the former is concerned with scale development, identifying the areas of greatest individual variability between individuals with a goal to distinguish those who have sleep disturbance or sleep-related impairment from those who do not, whereas, the latter is concerned with intraindividual differences in judgment across scenarios, with a goal to clarify what it means when a person says, ‘I had a good/ bad night’s sleep’. The application of IRT in the study of insomnia has been focused on scale development, which is diagnostic and predictive.^64,65^ In contrast, the application of choice-based conjoint analysis in this study is more revelatory and retrospective.

### Potential Clinical Implications

As the current study was the first attempt applying choice-based conjoint analysis to unpack the subjective meaning of sleep quality, we wish to be cautious in our extrapolation of what the findings might mean for clinical practice. However, if we were to speculate, the methodology described here has the potential to help us identify the specific factors that drive patient complaints of “poor sleep quality”, particularly in cases where “objective” assessments of sleep showed no conclusive finding of sleep disturbance. This could help clinicians to understand potential causes of “poor sleep quality” complaints of individual patients and accordingly narrow down areas that warrant treatment that may differ between patients.

Recent advances in digital technology have opened up numerous possibilities for eliciting information from patients. With a few tweaks in scenario sampling algorithms and data analysis approaches, the sleep quality judgment decision-making task like the one used in the current study could be run on smartphones, in combination with the recommended 2-week sleep diary assessment.^66^ Aided by corresponding computer applications, clinicians can be provided with diagnostics based on these sleep quality judgment data for personalizing the assessment and treatment plan, allowing the field to move yet another step closer toward patient-centred care^67–70^ and personalized medicine.^71^ Obviously, the automation of the diagnostic application would require research that shortens the elicitation procedure, perhaps by collecting a large number of trials from a variety of participants, so that new patients can be matched to this normative data using a small number of trials. Also, further research is needed to better understand whether factors such as circadian rhythm, day-to-day variability in sleep, use of sleep medication, social conventions (e.g., weekday/weekend distinction), and even weather (e.g., availability of sunshine) influence the sleep quality decision-making process, and if so, how.

## CONCLUSION

In conclusion, sleep quality judgments appear to be determined by not only what happened during sleep, but also what happens after the sleep period. Interventions that improve mood and functioning during the day may inadvertently also improve people’s subjective evaluation of sleep quality.

## SUPPLEMENTARY MATERIAL

Supplementary data are available at *SLEEP* online.

## FUNDING

ANS was supported by the ESRC (Grant number: ES/K004948/1). NT’s research is supported by the National Institute for Health Research, UK(Grant reference number: PB-PG-0213-30121).

## DISCLOSURE STATEMENT

All authors declared no conflict of interest

## Supplementary Material

Supplementary_Materials
