# Supplementary material for: What Sways People’s Judgment of Sleep Quality? A Quantitative Choice-Making Study With Good and Poor Sleepers
Source: Sleep. 2017 May 19;40(7):zsx091. doi: 10.1093/sleep/zsx091 (PMC5804994; doi:10.1093/sleep/zsx091)
Supplement: Supplementary_Materials [file zsx091_suppl_supplementary_materials.docx]

**Supplementary Materials/ Appendix 1**

The exact instructions on the computer screen read as follows:

“*On the following pages, you will be shown two scenarios per page, each inside its own box. Click on a box to read the scenario inside. You are allowed to read each scenario a maximum of two times.*

*The two scenarios can be quite similar to each other. Please read each carefully and importantly imagine yourself being the person who experienced the scenario described.*

*You can only read one scenario at a time so try to get an overall impression of a scenario as you read it. When you have established a clear picture of the scenarios in your mind, pick the scenario that you feel answers the question about sleep quality.*

*Use a select button to indicate your choice (the buttons will be available after you have read both scenarios). There is no time limitation, but please respond as quickly and accurately as possible*.”

**Supplementary Materials/ Appendix 2**

Ideally we would like to know the sleep quality of each of the stories, but because there is a very large number of possible stories (3 options ^16parameters^ x 5 options ^1 parameter^ = 215,233,605 stories), collecting choices for each pair of stories is not feasible. Instead, we applied the Markov chain Monte Carlo algorithm^72^ developed in computer science and statistics to solve the similar problem of summarising complex probability distributions. The MCMC algorithm takes samples from the complex distribution to provide an approximation of the distribution.

Our data were collected using the Markov Chain Monte Carlo with People method,^34^ which draws samples from people’s mental representations and is an application of the MCMC algorithm. Starting in an initial “state” (in our case, a scenario), the algorithm proceeds by first making a “proposal”, which is a randomly modified version of the initial state. With this state and proposal, in a fully automated computer program the MCMC algorithm decides whether the next state in the chain should be either the current state or the proposal, given that the probability of choosing the proposal is a ratio of the probabilities of the state and proposal [e.g., $p(x_{proposal})/(p\left( x_{state} \right)+p\left( x_{proposal} \right)]$. A long series of these sequential choices are made, which forms the “chain” of states. This resulting chain of states is a series of samples from the complex probability distribution.

MCMCP turns this fully automated algorithm into one in which people make decisions, instead of the machine. This is applied to explore people’s mental representations of subjective concepts, such as sleep quality. The computer executes most of the procedure as before, randomly proposing new states and keeping track of the chain. However, instead of letting the computer decide whether to transition to a new state, a participant makes the decision of whether to stay with the current state or transition to the proposal.

In our case, participants do this by viewing a pair of stories (i.e. the state and the proposal) and choosing which scenario better answers a question about sleep quality. The chosen scenario is the new state of the chain. Because people make these forced-choice decisions probabilistically [e.g. $m(x_{proposal})/(m\left( x_{state} \right)+m\left( x_{proposal} \right))]$, where $m(x)$ is the match of scenario $x$ to the question), we can use their decisions as a replacement for the decision function in the MCMC algorithm. The choices participants make in a long sequence of chained decisions will appear with probability equal to their relative match to the question: the best scenario should appear the most often, the second-best the second most often etc.

However in MCMCP, like in MCMC, care must be taken to discard the initial few states of the chain because they are heavily influenced by what the experimenter chose for the initial state (i.e., not the participant’s mental representation). The data discarded is called the “burn-in” (discussed in the Results section). Also, the states that are produced are auto-correlated, the effective sample size is smaller than the actual sample size. Hence, while the counts of the chosen stories are an estimate of people’s mental representation of sleep quality, we chose to analyze the individual choices that participants made in a logistic regression because these choices can be treated as independent, which gives us greater statistical power.

**Supplementary Materials/ Appendix 3**

Multiple chains of each group

| Good sleeper  (*n*= 50) | **Chain 1**  (12 trials)  Better night’s sleep | **Chain 2**  (12 trials)  Better night’s sleep | **Chain 5**  (12 trials)  Better night’s sleep | **Chain 6**  (12 trials)  Better night’s sleep | **Chain 9**  (12 trials)  Better night’s sleep | **Chain 10**  (12 trials)  Better night’s sleep |
| --- | --- | --- | --- | --- | --- | --- |
|  | **Chain 3**  (12 trials)  Worse night’s sleep | **Chain 4**  (12 trials)  Worse night’s sleep | **Chain 7**  (12 trials)  Worse night’s sleep | **Chain 8**  (12 trials)  Worse night’s sleep | **Chain 11**  (12 trials)  Worse night’s sleep | **Chain 12**  (12 trials)  Worse night’s sleep |

| Poor sleeper  (*n*= 50) | **Chain 1**  (12 trials)  Better night’s sleep | **Chain 2**  (12 trials)  Better night’s sleep | **Chain 5**  (12 trials)  Better night’s sleep | **Chain 6**  (12 trials)  Better night’s sleep | **Chain 9**  (12 trials)  Better night’s sleep | **Chain 10**  (12 trials)  Better night’s sleep |
| --- | --- | --- | --- | --- | --- | --- |
|  | **Chain 3**  (12 trials)  Worse night’s sleep | **Chain 4**  (12 trials)  Worse night’s sleep | **Chain 7**  (12 trials)  Worse night’s sleep | **Chain 8**  (12 trials)  Worse night’s sleep | **Chain 11**  (12 trials)  Worse night’s sleep | **Chain 12**  (12 trials)  Worse night’s sleep |

Notes. To break down the concept of chaining, we created the chains because we wanted to enable the algorithm to sample the different extreme scenarios (i.e., better vs. worse night’s sleep). This allows us to answer the question whether question type makes a difference in people’s judgement of sleep quality.

Each participant was only required to do 48 trials, which was not enough data to allow us to analyze participants individually. Therefore we chained participants together so that the last scenario chosen by one participant was used as one of the first choices for the next participant who was in that chain.

Given the sequential nature of the sampling, we also wanted to make the mutation process (see Figure 2) less obvious to the participants. By creating more chains, we could interleave them to masquerade the sequential nature of the sampling and make the choice-making task more varied and interesting to the participants to counteract cognitive fatigue. Finally, we created multiple groups of chains (good sleeper group: chains 1-4; chains 5-8; chains 9-12; poor sleeper group: chains 1-4; chains 5-8; chains 9-12). This was purely to help us speed up the data collection process, so we could run multiple participants at a time.

The removal of the data from the first participant of each chain was necessary because of the nature of the MCMCP sampling procedure, which has to start with particular arbitrary scenarios that neither represent a very good or a very poor night’s sleep. However, as participants along a chain made choices sequentially, the scenarios mutated towards the prototypical representations of good or poor night’s sleep (see Figure 2), allowing them to make choices between variations of these scenarios. That is why it is conventional to remove the “burn-in trials”, so choices made at the beginning of the MCMCP sampling procedure do not affect the overall results.
